# Clinical features of gastroenteritis during a large waterborne *Campylobacter* outbreak in Askøy, Norway

**DOI:** 10.1007/s15010-021-01652-3

**Published:** 2021-07-03

**Authors:** Knut Erik Emberland, K.-A. Wensaas, S. Litleskare, A. Iversen, K. Hanevik, N. Langeland, G. Rortveit

**Affiliations:** 1 Department of Global Public Health and Primary Care, University of Bergen, Bergen, Norway; 2 Research Unit for General Practice, NORCE Norwegian Research Centre, Bergen, Norway; 3 Department of Community Medicine, Askøy municipality, Norway; 4 Department of Clinical Science, University of Bergen, Bergen, Norway; 5 Norwegian National Advisory Unit On Tropical Infectious Diseases, Department of Medicine, Haukeland University Hospital, Bergen, Norway; 6 Department of Medicine, Haukeland University Hospital, Bergen, Norway

**Keywords:** Campylobacter infections, Disease outbreaks, Gastroenteritis, Waterborne diseases

## Abstract

**Purpose:**

Outbreaks of *Campylobacter* infection are common, but studies exploring the clinical features of acute illness in the outbreak setting are scarce in existing literature. The main purpose of the present study was to investigate the clinical features of self-reported acute illness in gastroenteritis cases during a large waterborne *Campylobacter* outbreak in Askøy municipality, Norway, in 2019.

**Methods:**

A web-based self-administered questionnaire, and invitation to participate was sent by the municipality of Askøy as text message to mobile phones using the municipality’s warning system to the inhabitants during the ongoing outbreak.

**Results:**

Out of 3624 participants, 749 (20.7%) were defined as cases, of which 177 (23.6%) reported severe gastroenteritis. The most common symptoms were loose stools (90.7%), abdominal pain (89.3%) and diarrhea (88.9%), whereas 63.8% reported fever, 50.2% joint pain and 14.2% bloody stools. Tiredness, a symptom non-specific to gastroenteritis, was the overall most common symptom (91.2%).

**Conclusion:**

About one in four of the cases reported symptoms consistent with severe gastroenteritis. We found more joint pain and less bloody stools than reported in published studies of laboratory confirmed campylobacteriosis cases. Tiredness was common in the current study, although rarely described in previous literature of acute illness in the outbreak setting.

**Supplementary Information:**

The online version contains supplementary material available at 10.1007/s15010-021-01652-3.

## Introduction

*Campylobacter* spp. is considered the most common bacterial cause of gastroenteritis worldwide, as well as in Europe and Norway [1–3]. Approximately 3000 cases are reported annually in Norway, of which more than 50% are acquired abroad. The domestically infected cases are either sporadic or associated with smaller outbreaks, most commonly waterborne [4, 5].

Common symptoms of gastroenteritis caused by *Campylobacter* include loose stools, diarrhea (≥ 3 loose stools in 24 h), nausea, vomiting, abdominal pain, bloody stools and fever, and the severity varies from mild and self-limiting symptoms (most common) to lethal disease [6–14]. Bloody stools and fever are considered markers of more severe infections [6, 13, 15, 16]. Several studies report symptoms and clinical features of campylobacteriosis, but these studies were predominantly published in the period from late 1970s to 2000, and based mainly on surveillance data or sporadic cases of laboratory confirmed infection [7–12, 15, 16]. Such cases represent a selected group that may differ from the total symptomatic population in the community [17, 18]. During large outbreaks of gastrointestinal infections, many cases are not tested and thus not registered. Most epidemiological studies on campylobacteriosis outbreaks aim to identify the source of the outbreak and rarely include detailed descriptions of clinical features.

In some cases, campylobacteriosis is complicated with joint symptoms, or post-infectious neuropathy or irritable bowel syndrome (IBS) [8, 19–23]. Antibiotics are usually not needed in treatment of campylobacteriosis but may be useful in patients with severe disease [6, 15, 16, 24–27]. The burden of symptoms during an outbreak, including the extent of more severe disease is difficult to investigate since research cannot be planned in advance. Hence, comprehensive baseline data from outbreaks are relatively rare.

In June 2019, there was a large community-wide waterborne *Campylobacter* outbreak in the island municipality Askøy (population 29,500) in Norway. The outbreak was detected on 6 June 2019, and the outbreak investigation team consisting of the municipality of Askøy and the National Institute of Public Health’s (NIPH) later concluded that the drinking water had been contaminated by *Campylobacter jejuni* sometime in late May 2019 [28]. The outbreak investigation findings were published in a report stating that more than 1500 inhabitants were ill during the outbreak [28]. Further, 67 patients were admitted to hospital and 2 deaths were related to the outbreak [28, 29]. Our group has long experience with research on clinical manifestations and complications of gastroenteritis in an outbreak setting [30, 31] and established a large cohort study within days after the outbreak was acknowledged. The primary aim of this study was to describe the clinical features of self-reported acute gastroenteritis in a *Campylobacter* outbreak setting. Secondary aims were to investigate factors associated with severe gastroenteritis.

## Methods

The current paper is based on data from the baseline survey out of totally four surveys in the Askøy *Campylobacter* Outbreak Study (ASCOS), a longitudinal cohort study following the outbreak. Households in Askøy received an invitation to participate in the study on 20 June 2019. Invitations and a webpage link to the survey were sent by the municipality of Askøy by one text message (SMS) to approximately 16,000 mobile phones using the municipality’s warning system, encouraging as many household members as possible to answer a questionnaire. Information was also presented in public meetings arranged by the municipality, on the municipality’s web site, and on posters in municipality and GP offices during the study period. Participants of all ages were included in the study. Consent from parents was needed for participants under the age of 16, and parents were asked to answer the questionnaire on behalf of younger children. Inclusion was closed on 1 July 2019.

**Fig. 1:**
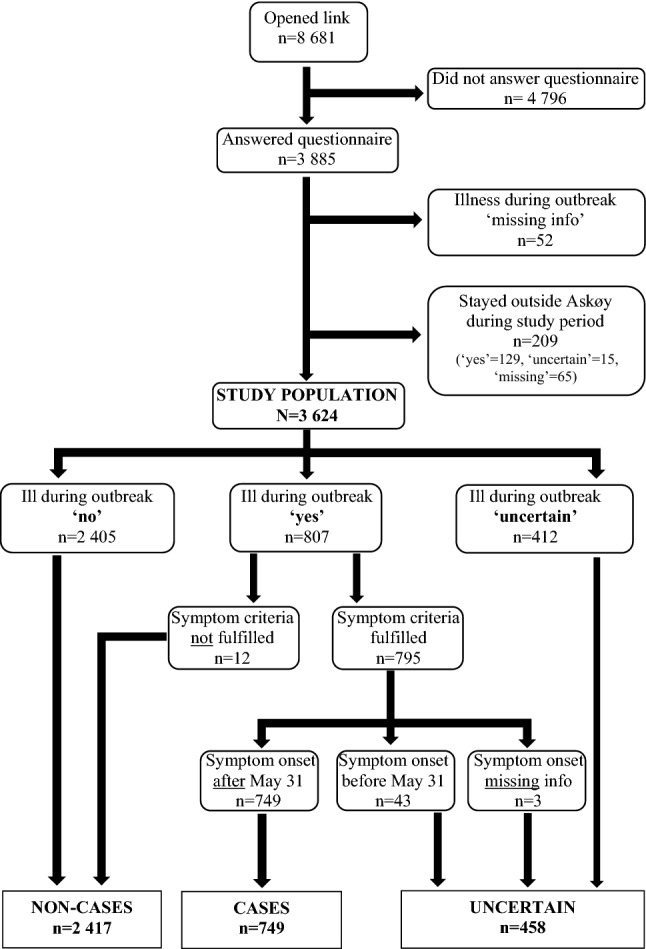
Flow chart of inclusion and exclusion of the study population, and of the cases, non-cases and the uncertain group, during the *Campylobacter* outbreak in Askøy

Participants were asked if they were ill during the outbreak. Participants responding ‘yes’ were further asked about the acute disease (symptoms, duration of disease and perceived severity), management (use of health care services and medication) and consequences of the disease (absence from work or school). Participants answering ‘no’ or ‘uncertain’ about acute illness did not receive these follow-up questions. Furthermore, all participants were asked if they had stayed continuously outside Askøy from 31 May up to the time for answering. The study population consists of all participants who answered the question whether they were ill during the outbreak, excluding those reporting that they had not been in Askøy during the outbreak (Fig. 1).

We defined a ‘case’ as a participant who reported being ill with gastrointestinal symptoms during the outbreak, with symptom onset in the study period, and who experienced at least one of the following symptoms: loose stools, diarrhea, bloody stools, abdominal pain, vomiting and nausea. A ‘non-case’ was a participant reporting not being ill during the outbreak *or* who reported being ill but did not fulfill the symptom criteria. Participants reporting that they were uncertain whether being ill during the outbreak *or* reported being ill and fulfilling the symptom criteria but with symptom onset either before the study period or missing, were assigned to the ‘uncertain’ group (Fig. 1). The participants were not asked whether they had submitted stool samples for laboratory verification of *Campylobacter* infection, as the aim of the study was to study self-reported gastroenteritis in the population during the *Campylobacter* outbreak.

Participants were asked to report ‘perceived severity’ at the worst time point during the acute illness, using a scale from 1 (well) to 9 (life-threatening illness). In addition, we defined the outcome ‘severe gastroenteritis’ as cases reporting diarrhea for ≥ 5 days *and* at least one of either fever for ≥ 2 days or bloody stools. Cases not fulfilling these criteria were defined as having ‘non-severe gastroenteritis’.

Duration of each of the following symptoms were specified by the predefined categories 1–2, 3–4 or 5–7 days, or 1–2 or > 2 weeks: loose stools, diarrhea, bloody stools, nausea, vomiting, abdominal pain, fever, joint pain, tiredness and other symptoms, in addition to total duration of illness (number of days). Questions whether still being ill and whether each symptom was still present at the time of answering the survey were also included. The study questionnaire furthermore included questions about age (number of years), sex, educational level (elementary school, high school, and college/university), employment situation (student/pupil, worker, self-employed, unemployed, on welfare, and pensioner), marital status (single, married, divorced/separated, and widow/widower), household total income in Norwegian kroner (< 250,000, 250,000–499,999, 500,000–749,999, 750,000–1,000,000, and > 1,000,000), self-reported previous diseases (diabetes, ulcerative colitis, Crohn’s disease, esophagitis, irritable bowel syndrome (IBS), celiac disease, peptic ulcer, anxiety, depression, and rheumatic disease), intake of glasses with tap water during the week prior to outbreak (0, 1–2, 3–5, > 5), intake of alcohol units during a normal week (number of units) and tobacco use (daily, sporadic, former daily smoker, and never smoked). Participants registered by their e-mail address, and were asked to voluntarily state their name, national identity number, telephone number and postal address for the purpose of follow-up studies and possibility/opportunity to be invited into adjacent research studies. All personally identifiable information were deidentified prior to analyses.

Two different categorical variables were made for age, with three (0–24, 25–54 and ≥ 55 years) and 10 categories (0–4, 5–14, 15–24, 25–34, 35–44, 45–54, 55–64, 65–74, 75–84 and ≥ 85 years), respectively. Duration of illness was categorized into 0–3, 4–7, 8–14 and ≥ 15 days. Alcohol units were categorized into the following six categories: 0, 1–2, 3–5, 6–9, 10–14 and ≥ 15 units per week. Tobacco use was dichotomized. Analyses of alcohol and tobacco use were restricted to participants ≥ 16 years. Analyses of the variables educational level, employment situation and marital status, were restricted to participants ≥ 18 years. There was a high proportion of missing data for the variables age and sex in the baseline survey (29% and 26%, respectively), but we were able to add data from the follow-up surveys for 580 and 507, respectively, giving a final of 13% missing age and 12% missing sex information in the study population.

The data were collected online using SuveyXact by Rambøll. All data have been stored, processed, and analyzed on the University of Bergen’s solution for secure processing of sensitive personal data in research (SAFE). The software R, StataSE 16.1 and Microsoft Excel for Windows 365 MSO have been used for processing and analyzing the data.

Descriptive statistics and Pearson’s *x*^2^-tests for associations were used to examine the distribution of different characteristics by two outcomes: (1) cases, non-cases and the uncertain group, and (2) cases with severe gastroenteritis vs. non-severe gastroenteritis. For the outcome severe gastroenteritis vs non-severe gastroenteritis, we further explored the associations by estimating relative risks (RRs) with 95% confidence intervals using a modified Poisson regression model [32], adjusting for sex and age. Distribution of symptoms, illness duration, management, and short-term consequences of the acute disease by sex and age were explored using descriptive statistics and Pearson’s *x*^2^-tests for associations. Level of statistical significance was set at *p* < 0.05.

## Results

During the study period, 8681 individuals accessed the web site, of which 3885 answered the questionnaire (Fig. 1). Of these, 261 were excluded because information about whether they had been ill was missing (n = 52) or because they had stayed outside Askøy (n = 209). In the study population of 3624 participants, 749 (20.7%) were cases, 2417 (66.7%) non-cases and 458 (12.6%) were uncertain. Tables 1 and 2 show the distribution of characteristics by cases, non-cases, and the uncertain group. The proportion of missing data for most variables, including sex and age, was highest in the uncertain group, followed by non-cases.

**Table 1:** Demographic characteristics of the study population, by cases with self-reported gastroenteritis, non-cases and the uncertain group, during the *Campylobacter* outbreak in Askøy

		All		Cases		Non-cases		Uncertain		*x*^2^
		*n*	*%*	*n*	*%*	*n*	*%*	*n*	*%*	*p*^*a*^
Total		3624	100	749	20.7*	2417	66.7*	458	12.6*	
Sex										0.174
	Male	1243	34.3	291	38.9	822	34.0	130	28.4	
	Female	1957	54.0	405	54.1	1291	53.4	261	57.0	
	Missing	424	11.7	53	7.1	304	12.6	67	14.6	
Age range (years)		1–91		1–82		1–91		1–81		
Age										< 0.01
	0–4	22	0.6	6	0.8	12	0.5	4	0.9	
	5–14	30	0.8	8	1.1	14	0.6	8	1.7	
	15–24	236	6.5	72	9.6	136	5.6	28	6.1	
	25–34	483	13.3	112	15.0	296	12.2	75	16.4	
	35–44	701	19.3	159	21.2	460	19.0	82	17.9	
	45–54	691	19.1	171	22.8	427	17.7	93	20.3	
	55–64	477	13.2	101	13.5	328	13.6	48	10.5	
	65–74	445	12.3	52	6.9	349	14.4	44	9.6	
	75–84	82	2.3	10	1.3	67	2.8	5	1.1	
	≥ 85	2	0.1	0	0.0	2	0.1	0	0.0	
	Missing	455	12.6	58	7.7	326	13.5	71	15.5	
Education level**										< 0.01
	Elementary school	175	4.9	47	6.4	102	4.3	26	5.9	
	High school	1192	33.6	299	40.9	748	31.5	145	32.7	
	University/college	1431	40.4	290	39.7	981	41.4	160	36.0	
	Missing	748	21.1	95	13.0	540	22.8	113	25.5	
Employment**										< 0.01
	Student/pupil	146	4.1	34	4.7	95	4.0	17	3.8	
	Worker	1879	53.0	459	62.8	1193	50.3	227	51.1	
	Self-employed	94	2.7	20	2.7	62	2.6	12	2.7	
	Unemployed	89	2.5	22	3.0	52	2.2	15	3.4	
	On welfare	177	5.0	55	7.5	95	4.0	27	6.1	
	Pensioner	423	11.9	50	6.8	336	14.2	37	8.3	
	Missing	738	20.8	91	12.4	538	22.7	109	24.5	
Houshold income										0.28
	< 250,000	86	2.4	23	3.1	51	2.1	12	2.6	
	250,000–499,999	393	10.8	89	11.9	254	10.5	50	10.9	
	500,000–749,999	609	16.8	130	17.4	388	16.1	91	19.9	
	750,000–1,000,000	683	18.8	171	22.8	437	18.1	75	16.4	
	> 1,000,000	900	24.8	191	25.5	621	25.7	88	19.2	
	missing	953	26.3	145	19.4	666	27.6	142	31.0	
Marital status**										< 0.01
	Single	440	12.4	131	17.9	251	10.6	58	13.1	
	Married/cohabitant	2152	60.7	459	62.8	1446	61.0	247	55.6	
	Divorced/separated	153	4.3	40	5.5	93	3.9	20	4.5	
	Widow/widower	61	1.7	9	1.2	45	1.9	7	1.6	
	Missing	740	20.9	92	12.6	536	22.6	112	25.2	

Distribution within characteristics is given by column unless stated by *

*Distribution by row, i.e., within study population

**Analyses restricted to participants ≥ 18 years

^a^*P* values from Pearson’s *x*^2^-test of association calculated from cross tables not including missing values and ‘uncertain group’

**Table 2:** Characteristics of the study population, by cases with self-reported gastroenteritis, non-cases, and the uncertain group, during the *Campylobacter* outbreak in Askøy

		All		Cases		Non-cases		Uncertain		*x*^2^
		*n*	*%*	*n*	*%*	*n*	*%*	*n*	*%*	*p*^*a*^
Total		3624	100	749	20.7*	2417	66.7*	458	12.6*	
Tap water (glasses/day)^b^										< 0.01
	0	225	6.2	21	2.8	183	7.6	21	4.6	
	1–2	963	26.6	158	21.1	664	27.5	141	30.8	
	3–5	1450	40.0	319	42.6	952	39.4	179	39.1	
	> 5	976	26.9	247	33.0	612	25.3	117	25.5	
	missing	10	0.3	4	0.5	6	0.2	0	0.0	
Alcohol (units/week)**^c^										< 0.01
	0	1396	39.1	328	44.6	894	37.5	174	39.0	
	1–2	999	28.0	191	26.0	682	28.6	126	28.3	
	3–5	539	15.1	100	13.6	375	15.7	64	14.3	
	6–9	191	5.4	45	6.1	126	5.3	20	4.5	
	10–14	81	2.3	15	2.0	57	2.4	9	2.0	
	≥ 15	26	0.7	4	0.5	18	0.8	4	0.9	
	missing	336	9.4	52	7.1	235	9.8	49	11.0	
Tobacco**										0.75
	Yes	1602	44.9	322	43.8	1076	45.1	204	45.7	
	No	1895	53.1	401	54.6	1260	52.8	234	52.5	
	Missing	71	2.0	12	1.6	51	2.1	8	1.8	
Previous diseases										
	None	1860	51.3	332	44.3	1329	55.0	199	43.4	< 0.01
	Diabetes	142	3.9	22	2.9	105	4.3	15	3.3	0.09
	Ulcerative colitis	50	1.4	6	0.8	34	1.4	10	2.2	0.20
	Crohn’s disease	16	0.4	4	0.5	7	0.3	5	1.1	0.32
	Oesophagitis	150	4.1	41	5.5	88	3.6	21	4.6	0.03
	Irritable bowel syndrome	318	8.8	76	10.1	187	7.7	55	12.0	0.04
	Celiac disease	35	1.0	12	1.6	17	0.7	6	1.3	0.02
	Peptic ulcer	93	2.6	24	3.2	51	2.1	18	3.9	0.09
	Anxiety	325	9.0	83	11.1	187	7.7	55	12.0	< 0.01
	Depression	344	9.5	89	11.9	207	8.6	48	10.5	< 0.01
	Rheumatic/inflammatory	207	5.7	54	7.2	121	5.0	32	7.0	0.02

Distribution within characteristics is given by column unless stated by *

*Distribution by row, i.e., within study population

**Analyses restricted to participants ≥ 16 years old

^a^*p* values from Pearson’s *x*^2^-test of association calculated from cross tables not including missing values and ‘uncertain group’

^b^Average daily number of tap water glasses during week before outbreak

^c^Units of alcohol during a normal week

**Fig. 2:**
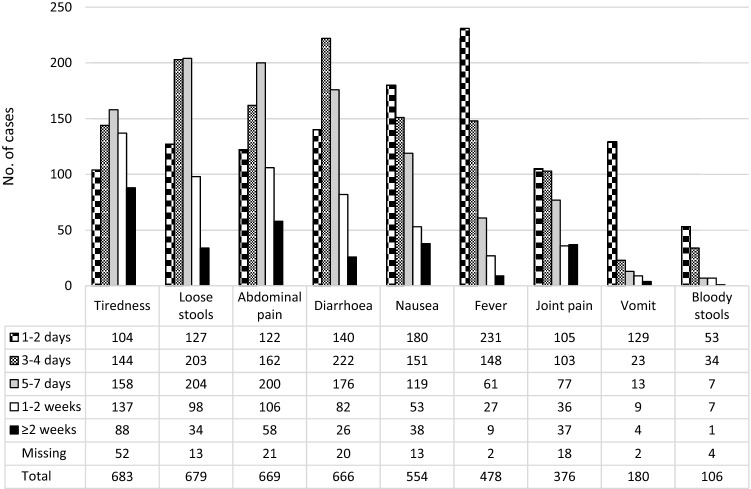
Duration of symptoms reported by cases with self-reported gastroenteritis, during the *Campylobacter* outbreak in Askøy. N = 749

The most common symptoms reported by the 749 cases were tiredness (91.2%), loose stools (90.7%), abdominal pain (89.3%) and diarrhea (88.9%) (Table 3 and Fig. 2). Bloody stools (14.2%) and vomiting (24.0%) were the least frequently reported of the listed symptoms. Nausea and joint pain were reported by 74.0% and 50.2%, respectively, whereas 63.8% of the cases reported fever. More women than men reported nausea (79.3% in females vs. 66.7% in males, *p* < 0.01), abdominal pain (91.4% in females vs. 87.3% in males, *p* = 0.02) and joint pain (54.1% in females vs. 45.4 in males, *p* = 0.02). Vomiting, fever, and tiredness were more commonly reported among the youngest and the oldest, compared to those aged 25–54 years, whereas diarrhea was most common in age category 25–54 years. Bloody stools were most frequently reported by cases in the age category 0–24 years (25.6%); however, none of these was under the age of 15 years. In age category 25–54 years, bloody stools were reported in 14.7%, and in 8.6% of those 55 years or older.

**Table 3:** Symptoms, management, and consequences of illness during the *Campylobacter* outbreak in Askøy, by age

Age category (years)		All		0–24		25–54		≥ 55		Missing		*x*^2^
		*n*	*%*	*n*	*%*	*n*	*%*	*n*	*%*	*n*	*%*	p**
Total		749	100	86	11.5*	442	59.0*	163	21.8*	58	7.7*	
Symptoms												
	Loose stools	679	90.7	77	89.5	408	92.3	144	88.3	50	86.2	0.73
	Diarrhea	666	88.9	70	81.4	408	92.3	138	84.7	50	86.2	0.04
	Bloody stools	106	14.2	22	25.6	65	14.7	14	8.6	5	8.6	< 0.01
	Nausea	554	74.0	71	82.6	321	72.6	120	73.6	42	72.4	0.12
	Vomit	180	24.0	31	36.0	93	21.0	40	24.5	16	27.6	0.01
	Abdominal pain	669	89.3	78	90.7	400	90.5	142	87.1	49	84.5	0.94
	Fever	478	63.8	69	80.2	271	61.3	107	65.6	31	53.4	< 0.01
	Joint pain	376	50.2	40	46.5	225	50.9	84	51.5	27	46.6	0.22
	Tiredness	683	91.2	82	95.3	396	89.6	151	92.6	54	93.1	< 0.01
Illness duration (days)												0.43
	0–3	120	16.0	13	15.1	75	17.0	16	9.8	16	27.6	
	4–7	316	42.2	40	46.5	196	44.3	53	32.5	27	46.6	
	8–14	171	22.8	22	25.6	108	24.4	33	20.2	8	13.8	
	≥ 15	33	4.4	6	7.0	16	3.6	10	6.1	1	1.7	
	Missing	109	14.6	5	5.8	47	10.6	51	31.3	6	10.3	
Consulted doctor		203	27.1	31	36.0	109	24.7	53	32.5	10	17.2	0.01
Hospitalized		33	4.4	3	3.5	16	3.6	12	7.4	2	3.4	0.12
Absence school/work		414	55.3	59	68.6	281	63.6	46	28.2	28	48.3	< 0.01
Medication												
	None	184	24.6	21	24.4	109	24.7	42	25.8	12	20.7	0.03
	Antibiotics	21	2.8	2	2.3	10	2.3	8	4.9	1	1.7	0.12
	Loperamide	93	12.4	7	8.1	49	11.1	35	21.5	2	3.4	< 0.01
	Tramadol	13	1.7	1	1.2	5	1.1	6	3.7	1	1.7	0.10
	Codeine + paracetamol	41	5.5	3	3.5	24	5.4	8	4.9	6	10.3	0.39
	Paracetamol	470	62.8	59	68.6	281	63.6	92	56.4	38	65.5	0.58
	NSAIDs	238	31.8	41	47.7	156	35.3	21	12.9	20	34.5	< 0.01
	Probiotics	167	22.3	17	19.8	101	22.9	35	21.5	14	24.1	0.02

Distribution within characteristics is given by column unless stated by *

*Distribution by row

^**^p value from Pearson’s *x*^2^-test of association calculated from cross tables not including missing values

Illness duration ranged from 0 to 24 days, with a median at 6 days (interquartile range: 4–9 days) (Table 3). Nine percent (n = 68) reported still being ill at the time of answering the survey (data not shown). Slightly more men than women reported illness duration of 0–3 days (17.9% vs. 13.3%, *p* = 0.02), 4–7 days (42.3% vs. 41.2%, *p* = 0.02), and ≥ 15 days (5.2% vs. 4.2%, *p* = 0.02), while an illness duration of 8–14 days was more common in women than men (27.7% vs. 17.5%, *p* = 0.02). No significant differences across age were observed for the illness duration distribution (Table 3).

Each symptom’s duration turned out to fit into one of to two main patterns (Fig. 2): symptoms with (1) an approximate bell-shaped distribution where most cases reported either 3–4 days or 4–7 days duration (tiredness, loose stools, abdominal pain and diarrhea), and (2) symptoms most frequently reported to last for either 1–2 days or 3–4 days with a subsequent decrease (nausea, fever, joint pain, vomiting and bloody stools). At the time of answering the survey, the proportions of cases reporting each symptom being ‘still present’ were: tiredness 15.9%, abdominal pain 9.0%, joint pain 8.5%, nausea 6.1%, diarrhea 6.0%, loose stools 5.9%, fever 1.3%, bloody stools 1.2%, and vomiting 0.5% (data not shown).

Paracetamol (62.8%) was the medication most frequently reported for treatment of the acute illness, followed by non-steroidal anti-inflammatory drugs (NSAIDs) (31.8%) (Table 3). Antibiotics use was reported by 2.8% (n = 21) of the cases, of which none were under the age of 15 years. Among the hospitalized cases, 30.3% (n = 10) reported antibiotics use, compared to 1.5% (n = 11) of those not hospitalized. Loperamide was reported by 12.4% (*n* = 93) of the cases, most commonly used by those aged ≥ 55 years (21.5%) and least common in age category 0–24 years (8.1%). No cases under the age of 15 years reported use of loperamide. No significant differences between the sexes were observed for consulting a doctor or hospitalization (data not shown), whereas for medication more women than men reported use of paracetamol (65.4% vs. 58.1%, *p* = 0.04).

Twenty-seven percent (*n* = 203) of the cases reported to have consulted a primary care doctor, which was more common among the youngest and oldest compared to the middle-aged cases (Table 3). Four percent had been admitted to hospital, most commonly in the age category ≥ 55 years (Table 3). No cases below 15 years of age reported being admitted to hospital.

Twenty-four percent of the cases (n = 177) fulfilled the definition of ‘severe gastroenteritis’, and Supplementary tables 1 and 2 show the distribution and characteristics of these patients. There were significant positive associations between the outcome ‘severe gastroenteritis’, and the reported perceived severity at the worst time point of the illness (Table 4). Furthermore, cases with severe gastroenteritis more often had been in contact with a primary care doctor or were hospitalized (Table 4).

**Table 4:** Health care seeking and perceived severity of illness during the *Campylobacter* outbreak in Askøy, by cases with severe or non-severe gastroenteritis

		All		Non-severe		Severe		*x*^2^
		*n*	*%*	*n*	*%*	*n*	*%*	*p*^*a*^
Total		749	100	572	76.4*	177	23.6*	
Contacted doctor								< 0.01
	Yes	203	27.1	117	20.5	86	48.6	
	No	539	72.0	450	78.7	89	50.3	
	Uncertain	5	0.7	3	0.5	2	1.1	
	Missing	2	0.3	2	0.3	0	0.0	
Hospitalized								0.01
	Yes	33	4.4	16	2.8	17	9.6	
	No	715	95.5	555	97.0	160	90.4	
	Missing	1	0.1	1	0.2	0	0.0	
Perceived severity^b^								< 0.01
	1 well	8	1.1	6	1.0	2	1.1	
	2	39	5.2	37	6.5	2	1.1	
	3	125	16.7	118	20.6	7	4.0	
	4	152	20.3	133	23.3	19	10.7	
	5	157	21.0	133	23.3	24	13.6	
	6	160	21.4	99	17.3	61	34.5	
	7	83	11.1	37	6.5	46	26.0	
	8	21	2.8	8	1.4	13	7.3	
	9 life threatening	4	0.5	1	0.2	3	1.7	

Distribution within characteristics is given by column unless stated by *

*Distribution by row

^a^*p* values from Pearson’s *x*^2^-test of association calculated from cross tables between cases with non-severe and severe gastroenteritis

^b^Self-reported perceived severity at worst time point of illness. Scale from 1 (well) to 9 (life threatening)

Compared to the others, cases with severe gastroenteritis more often reported drinking > 5 glasses of tap water (41.2% vs 30.4%, *p* = 0.02), previous peptic ulcer (13.6% vs 2.3%, *p* = 0.01) and previous depression (16.9% vs 10.3%, *p* = 0.02) (Supplementary table 2). In the adjusted regression analyses, previous depression (RR: 1.62, 95% CI 1.17–2.26) and previous peptic ulcer (RR: 1.73, 95% CI 1.00–3.00) remained significant (Table 5). Further, age 55–64 years (RR: 0.63, 95% CI 0.41–0.94) and 35–44 (RR: 0.53, 95% CI 0.36–0.78), were associated with a lowered risk of severe gastroenteritis as compared to the reference age category 45–54 years, although the RR for age 55–64 years was not significant in the unadjusted regression model.

**Table 5:** Severe gastroenteritis by characteristics, during the *Campylobacter* outbreak in Askøy. Unadjusted and adjusted relative risks (RR) with 95% confidence intervals (CIs)

		Unadjusted		Adjusted^a^	
		RR	CI	RR	CI
Sex					
	Male	1.20	0.93—1.6	1.33	1.03—1.72
	Female	Reference		Reference	
Age group (years)					
	0–4	1.02	0.32—3.22	1.23	0.34—4.41
	5–14	0.38	0.06—2.42	0.35	0.05—2.23
	15–24	0.89	0.59—1.36	0.77	0.50—1.19
	25–34	0.85	0.59—1.22	0.77	0.53—1.12
	35–44	0.54	0.36—0.80	0.53	0.36—0.78
	45–54	Reference		Reference	
	55–64	0.67	0.43—1.02	0.63	0.41—0.96
	65–74	0.65	0.37—1.14	0.63	0.36—1.09
	75–84	0.92	0.35—2.42	0.84	0.30—2.37
	≥ 85	NA		NA	
Tap water (glasses/day)^b^					
	0	0.83	0.34—2.06	0.75	0.31—1.78
	1–2	0.72	0.48—1.08	0.72	0.48—1.08
	3–5	Reference		Reference	
	> 5	1.29	0.98—1.71	1.29	0.97—1.70
Diseases					
	None	0.84	0.65—1.10		
	Diabetes	1.56	0.89—2.76		
	Ulcerative colitis	2.14	0.95—4.81		
	Crohn’s disease	1.06	0.19—5.81		
	Oesophagitis	1.37	0.86—2.19		
	Irritable bowel syndrome	1.26	0.86—1.84		
	Celiac disease	0.35	0.05—2.29		
	Peptic ulcer	2.00	1.27—3.16	1.73	1.00—3.00
	Anxiety	1.32	0.92—1.89		
	Depression	1.51	1.09—2.09	1.62	1.17—2.26
	Rheumatic/inflammatory	0.94	0.56—1.57		

^a^Adjusted for sex, age, intake of tap water, peptic ulcer and depression

^b^Average daily number of tap water glasses during week before outbreak

NA not applicable

## Discussion

The typical gastroenteritis cases during the *Campylobacter* outbreak in our study were adults experiencing illness lasting for 4–7 days, with diarrhea, abdominal pain, and tiredness as the most common symptoms. About one in two of the cases reported fever or joint pain, whereas bloody stools and vomiting were less common. One in four of the cases fulfilled our definition of severe gastroenteritis. Risk factors associated with severe gastroenteritis were having depression or peptic ulcer prior to the outbreak, in addition to high consumption of tap water. Approximately 1 in 4 had consulted a doctor, and 4% had been hospitalized.

A main strength of this study was that data were collected during the acute phase of a large outbreak, which increases statistical power and reduces recall bias considerably. This also constitutes a solid basis for follow-up studies of post-infectious complaints after the outbreak.

Invitations to participate in the study were sent by the municipality of Askøy as text message to mobile phones using the municipality’s warning system, an approach that had recently been used by the municipality and the Norwegian Institute of Public Health as part of their outbreak investigation [28]. Askøy municipality has 29,500 inhabitants, and not all could be reached by this approach. Information about age and sex were extracted from the national identity number, for those who had stated this. Using this procedure secured precise information for those who responded to this request, but resulted in missing data for those who did not want to give this information.

As many as 458 participants, 12.6% of the study population, were uncertain whether they had been ill with acute gastrointestinal infection during the outbreak. Many persons in this group were likely having incident diffuse symptoms of other causes, but the size of the groups suggests that a fraction represents the less severe end of the spectrum of *Campylobacter* infection. As this group was not asked questions about symptoms, we could not categorize them as neither cases or non-cases based on such information. The uncertain group had the greatest proportion of missing data for most variables, representing a group with more uncertain answers overall. Since the cases were not laboratory confirmed nor did we have variables to verify exposure to *Campylobacter*, such as detailed information on the drinking water supply, we could not investigate potential risk factors for developing campylobacteriosis during the outbreak.

Cases were not verified by a clinician’s diagnosis or by laboratory information. Using this population-based approach, we were able to investigate a broad spectrum of symptoms during the outbreak. However, this also introduces some limitations. By use of a self-administered online questionnaire, we defined a ‘case’ based on the participants’ self-reported information about their geographical presence, onset of illness and symptoms related to the outbreak. There exist no common, symptom-based definitions of campylobacteriosis or gastroenteritis that are widely used for research purposes. Thus, our case definition was a modification of case definitions used in previous studies [33–36].

Our definition of ‘severe gastroenteritis’ was based on existing literature [6, 13, 15, 16], as well as clinical experiences and expertise among members in the research group, and aimed to capture a set of symptoms which indicated a greater extent of both local inflammation in the bowels (diarrhea for ≥ 5 days or bloody stools) and more generalized disease (fever > 2 days). We observed an association between ‘severe gastroenteritis’, and perceived severity at the worst time point during the illness, and health care use, which to some extent suggests validity to the definition.

Population-based cohort studies describing the clinical features of *Campylobacter* infection during an ongoing outbreak, are scarce in existing literature. The proportion of children with acute gastroenteritis was lower in our study compared to previous studies of *Campylobacter* infections [9, 10]. Selection bias may have led to underrepresentation of the elderly and children. The latter is suggested by our previous finding of 17 patients under the age of 16 years in the study that characterized hospitalized patients during the same outbreak [29], whereas the present study found no hospitalized cases under the age of 15 years.

Our finding of diarrhea and abdominal pain as the most common, and bloody stools and vomiting as the least common symptoms of acute gastroenteritis in the outbreak setting, is in line with previous literature on *Campylobacter* infection [8–10, 14, 34, 37]. The proportion of cases reporting bloody stools were 14% in the present study, which is higher compared to the findings in two previous outbreak investigation studies: a population-based study of an outbreak in Røros, Norway (2%) [34], and in a study of cases included among patients seeking health care services (of which 16% were laboratory confirmed) during an outbreak in Finland (4%) [37]. The two previously published studies of the Askøy outbreak; NIPH’s population-based outbreak investigation study (6%) [34] and the study of hospitalized cases (9%), also found lower proportions of bloody stools than the present study [28, 29]. Higher proportions of bloody stools, ranging from 30 to 58%, are reported in a study of laboratory confirmed cases in general practice in the Netherlands [9], of sporadic notified cases in Norway [10], of laboratory confirmed cases aged 0–14 years in an outbreak in Greece [38], and notified cases in Australia, Canada and the United states [14]. The latter study also reported association between age and bloody stools, in line with our finding, although their proportions of bloody diarrhea among the youngest (59% in age < 5 years, 49% in 5–24 years) were higher than in our study (25.6% aged < 25 years, but none < 15 years). The reason for lower proportions of bloody stools reported in outbreak studies, including the present study, may be that they capture a broader scope of clinical features than represented by the laboratory confirmed cases. The corresponding low proportion of bloody stools observed in the study of hospitalized patients during the Askøy outbreak, can be explained by a possible lowered threshold for referral due to fatal outcome in the initial phase of the outbreak, thus leading to hospitalization of less severe cases [29]. However, virulence factors associated with bloody stools of the particular strain of *Campylobacter jejuni* cannot be ruled out [29].

Joint pain was more common in our study compared to a Norwegian study of sporadic campylobacteriosis from 1992 (50% vs. 27%) [10], but otherwise seems to be scarcely described in existing literature as a symptom during the acute phase of *Campylobacter* infection. Tiredness was not a case-defining symptom, but still the most frequently reported in our study. We could not find descriptions of tiredness in published studies, probably because it is unspecific to gastroenteritis or *Campylobacter* infection. However, documenting the baseline level of the symptom at the time of the outbreak is useful to follow-up studies of post-infectious complaints, and should perhaps be investigated further in future outbreaks. Our findings of more common joint pain and less bloody stools in these cases with self-reported gastroenteritis than previously reported in studies of laboratory confirmed *Campylobacter* cases, may reflect that the population-based approach may capture a broader spectrum of clinical features of acute gastroenteritis during in the *Campylobacter* outbreak setting.

Median duration of illness observed in our study (6 days) is in line with what is commonly reported in previous studies (5–6 days) [14, 34, 37], except for the Norwegian study of sporadic cases from 1992 reporting median 11 days duration [10].

A total of 3%, none under the age of 15 years, and 30% of the hospitalized cases in the current study received antibiotic treatment. The study of hospitalized patients during the same outbreak found that one in two of children and one in ten of adults received antibiotics [29]. Kapperud et al. reported in 1992 that 16% of 135 sporadic laboratory confirmed cases in Norway were treated with antibiotics [10], and White et al. 2019 reported an antibiotic treatment proportion of 35% in culture confirmed cases in Australia, Canada and the United States [14], although neither discriminated between hospitalized and non-hospitalized treatment proportions. Our finding of low antibiotic treatment proportion is concordant with the recommendations, and with a generally cautious policy regarding use of antibiotics in Norway [6, 24, 25, 39].

Risk factors associated with severe gastroenteritis were high consumption of tap water, having depression or peptic ulcer prior to the outbreak, whereas being in the age category 35–44 seemed to be protective. As the outbreak was waterborne, the association between high consumption of tap water and severe gastroenteritis probably indicates a dose–response relationship. Psychological comorbidity has previously been shown to increase susceptibility to develop infectious gastroenteritis [23], but bias may lead to reporting of more severe symptoms in cases with depression, as the symptom pressure can be perceived as more burdensome in this patient group. However, this effect should have been reduced because the outcome ‘severe gastroenteritis’ in these analyses was defined by reported symptoms rather than the cases’ own assessment of perceived illness severity. Peptic ulcer as a risk factor for severe illness is reasonable, as a gastrointestinal disease, and not least presumably often treated with anti-acidic medication which may cause vulnerability to a more severe illness.

## Conclusions

We present clinical features of self-reported acute gastroenteritis in a population during a large waterborne outbreak of *Campylobacter* infection. The most common symptoms were loose stools, abdominal pain, and diarrhea. About one in four of the cases reported symptoms consistent with severe gastroenteritis. Although not a gastroenteritis specific symptom, tiredness was the overall most common symptom, but is rarely described in previous studies of acute campylobacteriosis.

## Supplementary Information

Below is the link to the electronic supplementary material.

Supplementary file1 (DOCX 23 kb)

Supplementary file2 (DOCX 18 kb)

## Data Availability

The data underlying this article cannot be shared publicly due to limitations given by the ethical approval by the Regional Committee for Medical and Health Research Ethics.
