# Correction to: Clinical features of gastroenteritis during a large waterborne *Campylobacter* outbreak in Askøy, Norway

**DOI:** 10.1007/s15010-021-01746-y

**Published:** 2022-01-20

**Authors:** Knut Erik Emberland, K.-A. Wensaas, S. Litleskare, A. Iversen, K. Hanevik, N. Langeland, G. Rortveit

**Affiliations:** 1 Department of Global Public Health and Primary Care, University of Bergen, Bergen, Norway; 2 Research Unit for General Practice, NORCE Norwegian Research Centre, Bergen, Norway; 3 Department of Community Medicine, Askøy municipality, Norway; 4 Department of Clinical Science, University of Bergen, Bergen, Norway; 5 Norwegian National Advisory Unit On Tropical Infectious Diseases, Department of Medicine, Haukeland University Hospital, Bergen, Norway; 6 Department of Medicine, Haukeland University Hospital, Bergen, Norway

## Correction to: Infection 10.1007/s15010-021-01652-3

The original version of this article unfortunately contained mistakes.

Section “Results”, second paragraph, third sentence should read: “More women than men reported nausea (79.3% in females vs. 66.7% in males, *p* < 0.01), abdominal pain (91.4% in females vs. 87.3% in males, *p* = 0.02) and joint pain (54.1% in females vs. 45.4 in males, *p* = 0.02)” .

Section “Results”, third paragraph, third sentence should read: “Slightly more men than women reported illness duration of 0–3 days (17.9% vs. 13.3%, *p* = 0.02), 4–7 days (42.3% vs. 41.2%, *p* = 0.02), and ≥ 15 days (5.2% vs. 4.2%, *p* = 0.02), while an illness duration of 8–14 days was more common in women than men (27.7% vs. 17.5%, *p* = 0.02)”.

Section “Results”, fifth paragraph, sixth sentence should read: “No significant differences between the sexes were observed for consulting a doctor or hospitalisation (data not shown), whereas for medication more women than men reported use of paracetamol (65.4% vs. 58.1%, *p* = 0.04)”.

Section “Results”, eighth paragraph, second sentence should read: “In the adjusted regression analyses, previous depression (RR: 1.62, 95% CI 1.17–2.26) and previous peptic ulcer (RR: 1.73, 95% CI 1.00–3.00) remained significant (Table 5). Further, age 55–64 years (RR: 0.63, 95% CI 0.41–0.94) and 35–44 (RR: 0.53, 95% CI 0.36–0.78), were associated with a lowered risk of severe gastroenteritis as compared to the reference age category 45–54 years, although the RR for age 55–64 years was not significant in the unadjusted regression model” .

The presentation of Table 1, Table 5 and the Supplementary file 1 were incorrect. The corrected tables (Tables 1 and 5) are given below and the Supplementary file 1 has also been updated to reflect the corrections.

**Table 1:** Demographic characteristics of the study population, by cases with self-reported gastroenteritis, non-cases and the uncertain group, during the *Campylobacter* outbreak in Askøy

		All		Cases		Non-cases		Uncertain		*x*^2^
		*n*	*%*	*n*	*%*	*n*	*%*	*n*	*%*	*p*^*a*^
Total		3624	100	749	20.7*	2417	66.7*	458	12.6*	
Sex										0.174
	Male	1243	34.3	291	38.9	822	34.0	130	28.4	
	Female	1957	54.0	405	54.1	1291	53.4	261	57.0	
	Missing	424	11.7	53	7.1	304	12.6	67	14.6	
Age range (years)		1–91		1–82		1–91		1–81		
Age										< 0.01
	0–4	22	0.6	6	0.8	12	0.5	4	0.9	
	5–14	30	0.8	8	1.1	14	0.6	8	1.7	
	15–24	236	6.5	72	9.6	136	5.6	28	6.1	
	25–34	483	13.3	112	15.0	296	12.2	75	16.4	
	35–44	701	19.3	159	21.2	460	19.0	82	17.9	
	45–54	691	19.1	171	22.8	427	17.7	93	20.3	
	55–64	477	13.2	101	13.5	328	13.6	48	10.5	
	65–74	445	12.3	52	6.9	349	14.4	44	9.6	
	75–84	82	2.3	10	1.3	67	2.8	5	1.1	
	≥ 85	2	0.1	0	0.0	2	0.1	0	0.0	
	Missing	455	12.6	58	7.7	326	13.5	71	15.5	
Education level**										< 0.01
	Elementary school	175	4.9	47	6.4	102	4.3	26	5.9	
	High school	1192	33.6	299	40.9	748	31.5	145	32.7	
	University/college	1431	40.4	290	39.7	981	41.4	160	36.0	
	Missing	748	21.1	95	13.0	540	22.8	113	25.5	
Employment**										< 0.01
	Student/pupil	146	4.1	34	4.7	95	4.0	17	3.8	
	Worker	1879	53.0	459	62.8	1193	50.3	227	51.1	
	Self-employed	94	2.7	20	2.7	62	2.6	12	2.7	
	Unemployed	89	2.5	22	3.0	52	2.2	15	3.4	
	On welfare	177	5.0	55	7.5	95	4.0	27	6.1	
	Pensioner	423	11.9	50	6.8	336	14.2	37	8.3	
	Missing	738	20.8	91	12.4	538	22.7	109	24.5	
Houshold income										0.28
	< 250,000	86	2.4	23	3.1	51	2.1	12	2.6	
	250,000–499,999	393	10.8	89	11.9	254	10.5	50	10.9	
	500,000–749,999	609	16.8	130	17.4	388	16.1	91	19.9	
	750,000–1,000,000	683	18.8	171	22.8	437	18.1	75	16.4	
	> 1,000,000	900	24.8	191	25.5	621	25.7	88	19.2	
	missing	953	26.3	145	19.4	666	27.6	142	31.0	
Marital status**										< 0.01
	Single	440	12.4	131	17.9	251	10.6	58	13.1	
	Married/cohabitant	2152	60.7	459	62.8	1446	61.0	247	55.6	
	Divorced/separated	153	4.3	40	5.5	93	3.9	20	4.5	
	Widow/widower	61	1.7	9	1.2	45	1.9	7	1.6	
	Missing	740	20.9	92	12.6	536	22.6	112	25.2	

Distribution within characteristics is given by column unless stated by *

*Distribution by row, i.e., within study population

**Analyses restricted to participants ≥ 18 years

^a^*P* values from Pearson’s *x*^2^-test of association calculated from cross tables not including missing values and ‘uncertain group’

**Table 5:** Severe gastroenteritis by characteristics, during the *Campylobacter* outbreak in Askøy. Unadjusted and adjusted relative risks (RR) with 95% confidence intervals (CIs)

		Unadjusted		Adjusted^a^	
		RR	CI	RR	CI
Sex					
	Male	1.20	0.93—1.6	1.33	1.03—1.72
	Female	Reference		Reference	
Age group (years)					
	0–4	1.02	0.32—3.22	1.23	0.34—4.41
	5–14	0.38	0.06—2.42	0.35	0.05—2.23
	15–24	0.89	0.59—1.36	0.77	0.50—1.19
	25–34	0.85	0.59—1.22	0.77	0.53—1.12
	35–44	0.54	0.36—0.80	0.53	0.36—0.78
	45–54	Reference		Reference	
	55–64	0.67	0.43—1.02	0.63	0.41—0.96
	65–74	0.65	0.37—1.14	0.63	0.36—1.09
	75–84	0.92	0.35—2.42	0.84	0.30—2.37
	≥ 85	NA		NA	
Tap water (glasses/day)^b^					
	0	0.83	0.34—2.06	0.75	0.31—1.78
	1–2	0.72	0.48—1.08	0.72	0.48—1.08
	3–5	Reference		Reference	
	> 5	1.29	0.98—1.71	1.29	0.97—1.70
Diseases					
	None	0.84	0.65—1.10		
	Diabetes	1.56	0.89—2.76		
	Ulcerative colitis	2.14	0.95—4.81		
	Crohn’s disease	1.06	0.19—5.81		
	Oesophagitis	1.37	0.86—2.19		
	Irritable bowel syndrome	1.26	0.86—1.84		
	Celiac disease	0.35	0.05—2.29		
	Peptic ulcer	2.00	1.27—3.16	1.73	1.00—3.00
	Anxiety	1.32	0.92—1.89		
	Depression	1.51	1.09—2.09	1.62	1.17—2.26
	Rheumatic/inflammatory	0.94	0.56—1.57		

^a^Adjusted for sex, age, intake of tap water, peptic ulcer and depression

^b^Average daily number of tap water glasses during week before outbreak

NA not applicable

The original article has been corrected.

