# Supplementary material for: Clinical features of gastroenteritis during a large waterborne Campylobacter outbreak in Askøy, Norway
Source: Infection. 2021 Jul 3;50(2):343–54. doi: 10.1007/s15010-021-01652-3 (PMC8942940; doi:10.1007/s15010-021-01652-3)
Supplement: Supplementary file 1 — Supplementary file1 (DOCX 23 kb) [file 15010_2021_1652_MOESM1_ESM.docx]

**Supplementary table 1.** Demographic characteristics of cases, by severe and non-severe gastroenteritis.

|  |  | **Total** | | **Non-severe** | | **Severe** | | ***x*^2^** |
| --- | --- | --- | --- | --- | --- | --- | --- | --- |
|  |  | *n* | *%* | *n* | *%* | *n* | *%* | *p^b^* |
| **Total** | | 749 | 100 | 572 | 76.4* | 177 | 23.6* |  |
| **Sex** | |  |  |  |  |  |  | 0.165 |
|  | male | 291 | 38.9 | 210 | 36.7 | 81 | 45.8 |  |
|  | female | 405 | 54.1 | 311 | 54.4 | 94 | 53.1 |  |
|  | missing | 53 | 7.1 | 51 | 8.9 | 2 | 1.1 |  |
| **Age range** | | 1-82 | | 1-82 | | 1-79 | |  |
| **Age** | |  |  |  |  |  |  | 0.110 |
|  | 0-4 | 6 | 0.8 | 4 | 0.7 | 2 | 1.1 |  |
|  | 5-14 | 8 | 1.1 | 7 | 1.2 | 1 | 0.6 |  |
|  | 15-24 | 72 | 9.6 | 51 | 8.9 | 21 | 11.9 |  |
|  | 25-34 | 112 | 15.0 | 81 | 14.2 | 31 | 17.5 |  |
|  | 35-44 | 159 | 21.2 | 131 | 22.9 | 28 | 15.8 |  |
|  | 45-54 | 171 | 22.8 | 115 | 20.1 | 56 | 31.6 |  |
|  | 55-64 | 101 | 13.5 | 79 | 13.8 | 22 | 12.4 |  |
|  | 65-74 | 52 | 6.9 | 41 | 7.2 | 11 | 6.2 |  |
|  | 75-84 | 10 | 1.3 | 7 | 1.2 | 3 | 1.7 |  |
|  | ≥85 | 0 | 0.0 | 0 | 0.0 | 0 | 0.0 |  |
|  | missing | 58 | 7.7 | 56 | 9.8 | 2 | 1.1 |  |
| **Marital status**** | |  |  |  |  |  |  |  |
|  | single | 131 | 17.9 | 92 | 16.5 | 39 | 22.4 | 0.496 |
|  | married/cohabitant | 459 | 62.8 | 343 | 61.6 | 116 | 66.7 |  |
|  | divorced/separated | 40 | 5.5 | 31 | 5.6 | 9 | 5.2 |  |
|  | widow/widower | 9 | 1.2 | 8 | 1.4 | 1 | 0.6 |  |
|  | missing | 92 | 12.6 | 83 | 14.9 | 9 | 5.2 |  |
|  | total ≥18 yrs | 731 | 100.0 | 557 | 100.0 | 174 | 100.0 |  |
| **Education level**** | |  |  |  |  |  |  | 0.313 |
|  | elementary school | 47 | 6.4 | 36 | 6.5 | 11 | 6.3 |  |
|  | high school | 299 | 40.9 | 214 | 38.4 | 85 | 48.9 |  |
|  | university/college | 290 | 39.7 | 223 | 40.0 | 67 | 38.5 |  |
|  | missing | 95 | 13.0 | 84 | 15.1 | 11 | 6.3 |  |
|  | total ≥18 yrs | 731 | 100.0 | 557 | 100.0 | 174 | 100.0 |  |
| **Employment**** | |  |  |  |  |  |  | 0.059 |
|  | student/pupil | 34 | 4.7 | 22 | 3.9 | 12 | 6.9 |  |
|  | worker | 459 | 62.8 | 346 | 62.1 | 113 | 64.9 |  |
|  | self employed | 20 | 2.7 | 16 | 2.9 | 4 | 2.3 |  |
|  | unemployed | 22 | 3.0 | 16 | 2.9 | 6 | 3.4 |  |
|  | on welfare | 55 | 7.5 | 33 | 5.9 | 22 | 12.6 |  |
|  | pensioner | 50 | 6.8 | 42 | 7.5 | 8 | 4.6 |  |
|  | missing | 91 | 12.4 | 82 | 14.7 | 9 | 5.2 |  |
|  | total ≥18 yrs | 731 | 100.0 | 557 | 100.0 | 174 | 100.0 |  |
| **Household income** | |  |  |  |  |  |  | 0.441 |
|  | < 250000 | 23 | 3.1 | 15 | 2.6 | 8 | 4.5 |  |
|  | 250000-499999 | 89 | 11.9 | 71 | 12.4 | 18 | 10.2 |  |
|  | 500000-749999 | 130 | 17.4 | 91 | 15.9 | 39 | 22.0 |  |
|  | 750000-1000000 | 171 | 22.8 | 129 | 22.6 | 42 | 23.7 |  |
|  | > 1000000 | 191 | 25.5 | 142 | 24.8 | 49 | 27.7 |  |
|  | missing | 145 | 19.4 | 124 | 21.7 | 21 | 11.9 |  |
| Distribution within characteristics is given by column unless stated by *.  * Distribution by row.  ** Analyses restricted to participants ≥18 years old.  ^a^ P-values from *x*^2^-test of association calculated from cross tables that do not include missing values. | | | | | | | | |
|  |  |  |  |  |  |  |  |  |
