# Supplementary material for: Clinical features of gastroenteritis during a large waterborne Campylobacter outbreak in Askøy, Norway
Source: Infection. 2021 Jul 3;50(2):343–54. doi: 10.1007/s15010-021-01652-3 (PMC8942940; doi:10.1007/s15010-021-01652-3)
Supplement: Supplementary file 2 — Supplementary file2 (DOCX 18 kb) [file 15010_2021_1652_MOESM2_ESM.docx]

| **Supplementary table 2.** Characteristics of cases with self-reported gastroenteritis, by severe and non-severe gastroenteritis, during the campylobacter outbreak in Askøy. | | | | | | | | |
| --- | --- | --- | --- | --- | --- | --- | --- | --- |
|  |  | All | | Non-severe | | Severe | | *x*^2^ |
|  |  | *n* | *%* | *n* | *%* | *n* | *%* | *p^a^* |
| Total | | 749 | 100 | 572 | 76.4 | 177 | 23.6 |  |
| Tap water (glasses/day)^b^ | |  |  |  |  |  |  | 0.02 |
|  | 0 | 21 | 2.8 | 17 | 3.0 | 4 | 2.3 |  |
|  | 1-2 | 158 | 21.1 | 132 | 23.1 | 26 | 14.7 |  |
|  | 3-5 | 319 | 42.6 | 246 | 43.0 | 73 | 41.2 |  |
|  | >5 | 247 | 33.0 | 174 | 30.4 | 73 | 41.2 |  |
|  | missing | 4 | 0.5 | 3 | 0.5 | 1 | 0.6 |  |
| Alcohol (units/week)**^c^ | |  |  |  |  |  |  | 0.44 |
|  | 0 | 328 | 44.6 | 255 | 45.5 | 73 | 42.0 |  |
|  | 1-2 | 191 | 26.0 | 151 | 26.9 | 40 | 23.0 |  |
|  | 3-5 | 100 | 13.6 | 75 | 13.4 | 25 | 14.4 |  |
|  | 6-9 | 45 | 6.1 | 30 | 5.3 | 15 | 8.6 |  |
|  | 10-14 | 15 | 2.0 | 11 | 2.0 | 4 | 2.3 |  |
|  | ≥15 | 4 | 0.5 | 4 | 0.7 | 0 | 0.0 |  |
|  | missing | 52 | 7.1 | 35 | 6.2 | 17 | 9.8 |  |
| Tobacco** | |  |  |  |  |  |  | 0.23 |
|  | yes | 322 | 43.8 | 240 | 42.8 | 82 | 47.1 |  |
|  | no | 401 | 54.6 | 314 | 56.0 | 87 | 50.0 |  |
|  | missing | 12 | 1.6 | 7 | 1.2 | 5 | 2.9 |  |
| Previous diseases | |  |  |  |  |  |  |  |
|  | none | 332 | 44.3 | 261 | 45.6 | 71 | 40.1 | 0.20 |
|  | diabetes | 22 | 2.9 | 14 | 2.4 | 8 | 4.5 | 0.15 |
|  | ulcerative colitis | 6 | 0.8 | 3 | 0.5 | 3 | 1.7 | 0.13 |
|  | Crohns disease | 4 | 0.5 | 3 | 0.5 | 1 | 0.6 | 0.10 |
|  | oesophagitis | 41 | 5.5 | 28 | 4.9 | 13 | 7.3 | 0.21 |
|  | IBS | 76 | 10.1 | 54 | 9.4 | 22 | 12.4 | 0.25 |
|  | celiac disease | 12 | 1.6 | 11 | 1.9 | 1 | 0.6 | 0.21 |
|  | peptic ulcer | 37 | 4.9 | 13 | 2.3 | 24 | 13.6 | 0.01 |
|  | anxiety | 83 | 11.1 | 58 | 10.1 | 25 | 14.1 | 0.14 |
|  | depression | 89 | 11.9 | 59 | 10.3 | 30 | 16.9 | 0.02 |
|  | rheumatic/inflammatory | 54 | 7.2 | 42 | 7.3 | 12 | 6.8 | 0.80 |
| Distribution within characteristics is given by column unless stated by *.  * Distribution by row.  ** Analyses restricted to participants ≥16 years old.  ^a^ P-values from Pearson’s *x*^2^-test of association calculated from cross tables that do not include missing values.  b Average daily number of tap water glasses during week before outbreak.  c Units alcohol during a normal week. | | | | | | | | |
